# Focal adhesion kinase overexpression and its impact on human osteosarcoma

**DOI:** 10.18632/oncotarget.5044

**Published:** 2015-09-03

**Authors:** Ke Ren, Xiao Lu, Nan Yao, Yong Chen, Aizhen Yang, Hui Chen, Jian Zhang, Sujia Wu, Xin Shi, Chen Wang, Xiaoliang Sun

**Affiliations:** ^1^ Department of Orthopedics, The Third Affiliated Hospital of Soochow University, The First People's Hospital of Changzhou, Changzhou 213003, Jiangsu Province, P.R.China; ^2^ Center Laboratory of Cancer Center, The Jingdu hospital of Nanjing, Nanjing 210002, Jiangsu Province, P.R.China; ^3^ Laboratory of Translational Medicine, Jiangsu Province Academy of Traditional Chinese Medicine, Nanjing 210028, Jiangsu Province, P.R.China; ^4^ Jinling Hospital, Department of Orthopedics, Nanjing University, School of Medicine, Nanjing 210002, Jiangsu Province, P.R.China; ^5^ Department of Orthopaedics, Zhongda Hospital, Southeast University, Nanjing 210009, Jiangsu Province, P.R.China

**Keywords:** osteosarcoma, focal adhesion kinase, prognosis, migration, invasion

## Abstract

Focal adhesion kinase (FAK) has been implicated in tumorigenesis in various malignancies. We sought to examine the expression patterns of FAK and the activated form, phosphorylated FAK (pFAK), in human osteosarcoma and to investigate the correlation of FAK expression with clinicopathologic parameters and prognosis. In addition, the functional consequence of manipulating the FAK protein level was investigated in human osteosarcoma cell lines. Immunohistochemical staining was used to detect FAK and pFAK in pathologic archived materials from 113 patients with primary osteosarcoma. Kaplan-Meier survival and Cox regression analyses were performed to evaluate the prognoses. The role of FAK in the cytological behavior of MG63 and 143B human osteosarcoma cell lines was studied via FAK protein knock down with siRNA. Cell proliferation, migration, invasiveness and apoptosis were assessed using the CCK8, Transwell and Annexin V/PI staining methods. Both FAK and pFAK were overexpressed in osteosarcoma. There were significant differences in overall survival between the FAK-/pFAK- and FAK+/pFAK- groups (*P* = 0.016), the FAK+/pFAK- and FAK+/pFAK+ groups (*P* = 0.012) and the FAK-/pFAK- and FAK+/pFAK+ groups (*P* < 0.001). There were similar differences in metastasis-free survival between groups. The Cox proportional hazards analysis showed that the FAK expression profile was an independent indicator of both overall and metastasis-free survival. siRNA-based knockdown of FAK not only dramatically reduced the migration and invasion of MG63 and 143B cells, but also had a distinct effect on osteosarcoma cell proliferation and apoptosis. These results collectively suggest that FAK overexpression and phosphorylation might predict more aggressive biologic behavior in osteosarcoma and may be an independent predictor of poor prognosis.

## INTRODUCTION

Osteosarcoma is the most common malignant tumor in bone and leads to a large number of cancer-related deaths in children and young adults, mainly due to the development of lung metastases [1–4]. The use of neoadjuvant chemotherapy began in the 1970s and has increased the five-year disease-free survival rate from approximately 20% to 60% [2], however, there have been only minimal improvements in the prognosis of osteosarcoma patients in the last two decades. In particular, patients with osteosarcoma lung metastases often have poor long-term survival given that more than 80% of these patients relapse within one year after surgical excision of lung metastases. Therefore, it is of great importance to explore the mechanisms of osteosarcoma development in order to provide a basis for new therapies as alternatives to traditional chemotherapy and surgery.

There are a limited number of predictors for patient prognosis in osteosarcoma. Most treatment strategies and prognostic information have only been based on the presence or absence of distant metastases at diagnosis and the response to neoadjuvant chemotherapy [5–6]. Other prognostic determinants have been insufficient to allow stratification of therapy, therefore, it is necessary to find novel prognostic indicators to stratify patients into low- and high-risk groups at initial diagnosis and to possibly indicate a role for effective targeted therapeutic agents [6–8].

Focal adhesion kinase (FAK) is a cytoplasmic protein tyrosine kinase that is localized at cell focal adhesion contacts and adhesion sites [9]. It has been proposed that the major substrate of FAK is FAK itself [10–11]. When cells adhere to fibronectin, FAK becomes autophosphorylated on tyrosine 397. This causes a second tyrosine kinase, called “src,” to become physically associated with FAK. Src then phosphorylates FAK on tyrosine 925, which leads to the recruitment of Grb-2 (an adapter protein) and SOS (a regulator of ras-type GTPases) to the FAK signaling complex [10, 12]. As a result, phosphorylation of FAK at Tyr397 dictates its function in response to integrin-mediated cell adhesion, migration, invasion and antiapoptosis, as well as growth factor–stimulated cell proliferation, as described in previous reports [13–14].

A few studies have demonstrated an up-regulation of FAK expression during the transformational process in which normal tissue is invaded by cancer after the stage of *in situ* carcinoma, suggesting that up-regulation of FAK might be an early event in carcinogenesis [15–17]. FAK overexpression has been reported as an independent prognostic factor for various types of cancers, including ovarian, esophagus and colon [15, 18–19]. These mechanistic and clinical findings indicate that FAK plays an important role in tumor cell activity and disease progression. So far, there have only been a few reports linking FAK to osteosarcoma. In the current study, the association between FAK, different degrees of FAK phosphorylation (regarded as different levels of one factor) and the clinicopathological features and survival of patients with osteosarcoma were analyzed to evaluate the clinical significance of FAK as a molecular indicator of osteosarcoma prognosis.

## RESULTS

### Expression and cellular distribution of FAK and pFAK in osteosarcoma

The patients in this study had a median follow-up period of 56 months (range 7 to 160 months) and the cumulative five-year overall survival rate was 51.1%. During follow-up, 77 (68.1%) patients died of tumor-related causes and 17 (15.0%) and 79 (69.9%) patients had local recurrences and distant metastases, respectively. Two patients had local recurrences alone and 15 patients experienced concurrent local recurrence and metastases. One patient was alive after undergoing wide excision of solitary metastases and one patient, who had local recurrence only, was alive and disease-free after undergoing amputation. The expression of FAK and pFAK was assessed in a cohort of osteosarcoma patients, including 71 (62.83%) males and 42 (37.17%) females, with an overall median age of 20.3 years (range 5–56 years).

**Figure 1:**
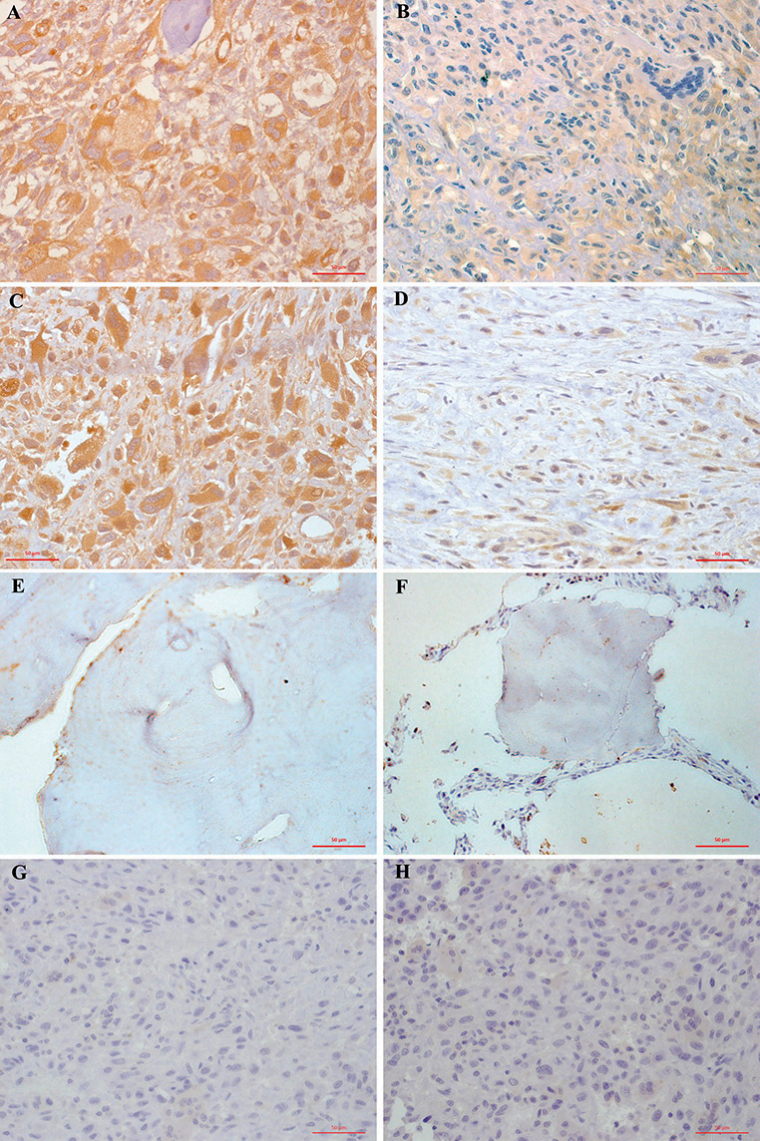
Immunohistochemical staining of FAK (A, B, E, G) and pFAK (C, D, F, H) proteins in osteosarcoma and normal cancellous bone tissues **A.** FAK was overexpressed in 61.95% (70/113) of osteosarcoma cells. Tumor cells exhibited cytoplasmic and sometimes membranous immunoreactivity for FAK. **B.** Low FAK immunohistochemical staining was shown in 43 osteosarcoma patients. **C.** pFAK was mainly expressed in the cytoplasm of osteosarcoma cells in 37.17% (42/113) of cases. **D.** Low phospho-FAK immunohistochemical staining pFAK was shown in 71 osteosarcoma patients. **E.** No FAK immunohistochemical staining was observed in normal cancellous bone tissues. **F.** No pFAK immunohistochemical staining pFAK was observed in normal cancellous bone tissues. **G.** FAK immunohistochemical staining was not present in negative controls. **H.** pFAK immunohistochemical staining was not present in negative controls. Results shown here are representative images, ×400 magnification.

The expression and cellular distribution of FAK and pFAK in the 113 human osteosarcoma specimens and 22 normal cancellous bone tissues were examined using immunohistochemical staining. Staining results are shown in Figure 1 and varied in the intensity and percentage of positive tumor cells. FAK was overexpressed in 61.95% (70/113) of osteosarcoma specimens with unequal intensity. Tumor cells exhibited cytoplasmic and sometimes membranous immunoreactivity for FAK (Figure 1A–1B). pFAK was expressed, mainly in the cytoplasm of osteosarcoma cells, in 37.17% (42/113) of cases (Figure 1C–1D). No overexpression staining of anti-FAK and anti-pFAK antibodies was observed in normal cancellous bone tissues (Figure 1E–1F) or in negative controls (Figure 1G–1H).

### Correlation of high FAK and pFAK expression with the clinicopathological characteristics of stage II extremity osteosarcoma

Expression of FAK and pFAK was assessed by immunohistochemical staining in sections from 113 osteosarcoma cases. The χ^2^ test (Table 1) showed no significant statistical correlation of FAK or pFAK immunostaining with age, gender, tumor location, AJCC surgical stage, surgical type (amputation or limb salvage surgery) or histological response to pre-operative chemotherapy (tumor necrosis rate) (*P* < 0.05), suggesting that these variables are not associated with the expression of FAK and/or its phosphorylation status.

**Table 1:** The association of clinicopathological data and FAK expression profiles in patients with stage II AJCC stage extremity osteosarcoma

Clinicopathological data	*n*	FAK-/pFAK- (%)	FAK+/pFAK- (%)	FAK+/pFAK+ (%)	*χ*^2^^a^	*P* value
Age (years)						
<18	50	19 (38.0)	10 (20.0)	21 (42.0)	1.390	0.518
≥18	63	24 (38.1)	18 (28.6)	21 (33.3)		
Gender						
Male	71	28 (39.4)	15 (21.1)	28 (39.4)	1.389	0.531
Female	42	15 (35.7)	13 (31.0)	14 (33.3)		
Clinical stage						
II A	45	20 (44.4)	11 (24.4)	14 (31.1)	1.544	0.506
II B	68	23 (33.8)	17 (25.0)	28 (41.2)		
Surgery						
Amputation	36	14 (38.9)	11 (30.6)	11 (30.6)	1.343	0.506
Limb salvage	77	29 (37.7)	17 (22.1)	31 (40.3)		
Histological response^b^						
<90%	62	24 (38.7)	16 (25.8)	22 (35.5)	0.179	0.914
≥90%	51	19 (37.3)	12 (23.5)	20 (39.2)		
Tumor site						
Femur	57	24 (42.1)	14 (24.6)	19 (33.3)	2.218	0.709
Tibia or fibula	35	10 (28.6)	10 (28.6)	15 (42.9)		
Other	21	9 (42.9)	4 (19.0)	8 (38.1)		

a Pearson *χ*^2^ test (two-sided)

b Tumor necrosis rate

### Prognostic value of FAK and pFAK overexpression

The correlation of FAK and pFAK expression with survival time and metastases was assessed in order to further examine the functional relevance of FAK overexpression and its phosphorylation in a subset of osteosarcoma patients. The patients were divided into three groups based on the FAK expression profiles, which were defined as the presence or absence of FAK overexpression and FAK phosphorylation: (A) FAK-/pFAK-, (B) FAK+/pFAK-, (C) FAK+/pFAK+. The median overall survival (OS) time was 86 months for group A, 56 months for group B and 39 months for group C. The metastasis-free median survival (MFS) time was 69 months for group A, 45 months for group B and 24 months for group C. Furthermore, the five-year overall survival rate was 78.4% for group A as compared with 44.9% and 25.8% for groups B and C, respectively. The five-year metastasis-free survival rate was 55.0% for group A as compared with 27.6% and 16.9% for groups B and C, respectively. These results suggest that high FAK expression and FAK phosphorylation were associated with decreased overall survival and metastasis-free survival times.

**Figure 2:**
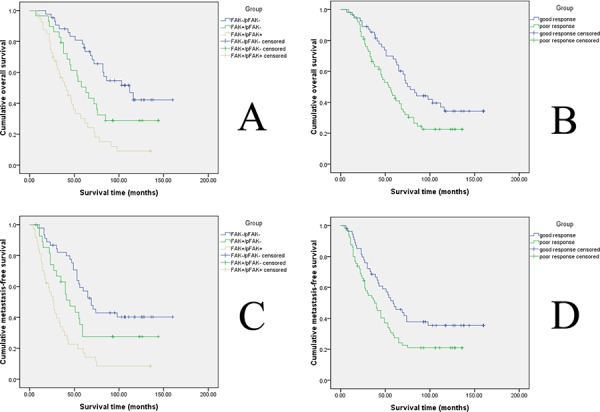
Kaplan–Meier analysis of overall survival and metastasis-free survival for patients with different FAK expression levels and for patients with different histological responses to pre-operative chemotherapy **A.** Kaplan–Meier analysis of overall survival in osteosarcoma. The blue line represents the FAK-/pFAK- group, the green line represents the FAK+/pFAK- group, and the brown line represents the FAK+/pFAK+ group. **B.** Kaplan–Meier analysis of metastasis-free survival in osteosarcoma. The blue line represents the FAK-/pFAK- group, the green line represents the FAK+/pFAK- group, and the brown line represents the FAK+/pFAK+ group. **C.** Kaplan–Meier analysis of overall survival in osteosarcoma. The green line represents patients with a good histological response to pre-operative chemotherapy and the blue line represents patients with a poor histological response to pre-operative chemotherapy. **D.** Kaplan–Meier analysis of metastasis-free survival in osteosarcoma. The green line represents patients with a good histological response to pre-operative chemotherapy and the blue line represents patients with a poor histological response to pre-operative chemotherapy.

Since survival in patients with osteosarcoma has been associated with several clinicopathological variables, univariate analysis was performed to identify the patient characteristics correlated with survival. The Kaplan-Meier survival analysis model indicated that the FAK expression profile (power = 1.000 for OS, power = 1.000 for MFS) and histological response to pre-operative chemotherapy (power = 0.883 for OS, power = 0.776 for MFS) were associated with both overall survival (Figure 2A–2B) and metastasis-free survival(Figure 2C–2D), however, no such association was found for gender, patient age, AJCC surgical stage, tumor location or surgical type (Table 2). Postoperative overall survival times were then analyzed (log-rank test) by pairwise comparison for each stratum for FAK expression profiles. There were significant differences between groups A and B (*P* = 0.016), groups B and C (*P* = 0.012), and groups A and C (*P* < 0.001). Group C was characterized by a significant decrease in overall survival rate compared with groups B and A and overall survival in group B was relatively worse than in group A. Similarly, the metastasis-free survival times were significantly different between groups A and B (*P* = 0.042), groups B and C (*P* = 0.001) and groups A and C (*P* < 0.001). Group C had the poorest metastasis-free survival rate among the three groups, while group A had the best prognosis.

**Table 2:** Univariate analyses of factors associated with OS and MFS

Variable	OS		*P* value	MFS		*P* value
	Median survival months	95% CI		Median survival months	95% CI	
Femur			**0.630**			**0.587**
Tibia or fibula	61	48–74		45	35–55	
Other	62	38–86		39	15–63	
Femur	65	29–101		43	13–73	
Age, years			**0.576**			**0.444**
< 18	63	36–90		51	24–78	
≥ 18	61	51–71		41	35–47	
Gender			**0.645**			**0.565**
Male	63	53–73		43	37–49	
Female	54	35–73		38	18–58	
Surgery			**0.915**			**0.803**
Amputation	63	50–76		51	40–62	
Limb salvage	61	45–77		40	29–51	
AJCC surgical stage			**0.916**			**0.778**
II A	62	40–84		43	23–63	
II B	59	46–72		41	35–47	
Histological response			**0.032**			**0.021**
Poor	49	36–62		35	23–47	
Good	72	61–83		54	40–68	
FAK expression			**<0.001**			**<0.001**
FAK−/pFAK−	86	52–120		69	54–84	
FAK+/pFAK−	56	48–64		45	37–53	
FAK+/pFAK+	39	27–51		24	18–30	

Abbreviations: CI, confidence interval; OS, overall survival; MFS, metastasis-free survival; FAK, focal adhesion kinase

All significant variables were assessed using the Cox proportional hazards model, a form of multivariate regression analysis, to further examine the relationship between independent variables and overall or metastasis-free survival. This analysis showed that the FAK expression profiles (power = 1.000) and histological response to pre-operative chemotherapy (power = 0.883) were independent indicators of overall survival (Table 3). Similarly, FAK expression profiles (power = 1.000) and histological response to pre-operative chemotherapy (power = 0.776) also seemed to be independent indicators of metastasis-free survival, except when the FAK expression profiles of group B were compared with those of group C (Table 3). Therefore, these results verify that FAK overexpression and FAK phosphorylation are correlated with low overall survival and metastasis-free survival, but suggest that other factors are independent of FAK expression profiles.

**Table 3:** Multivariate analysis of factors associated with OS and MFS

	Hazard ratio	95% CI	*P* value
OS			
FAK expression			
FAK+/pFAK+ versus FAK−/pFAK−	4.947	2.843–8.606	<0.001
FAK+/pFAK− versus FAK−/pFAK−	2.072	1.141–3.761	0.017
Histological response to pre-operative chemotherapy			
poor versus good	2.047	1.294–3.237	0.024
MFS			
FAK expression			
FAK+/pFAK+ versus FAK−/pFAK−	4.915	2.907–8.311	<0.001
FAK+/pFAK− versus FAK−/pFAK−	1.785	0.992–3.213	0.053
Histological response to pre-operative chemotherapy			
poor versus good	2.058	1.321–3.205	0.001

Variables were adopted for their prognostic significance by univariate analysis with enter-stepwise selection (*P* < 0.05).

Abbreviations: CI, confidence interval; OS, overall survival; MFS, metastasis-free survival; FAK, focal adhesion kinase

### FAK siRNA transfection reduced the expression of FAK and pFAK

**Figure 3:**
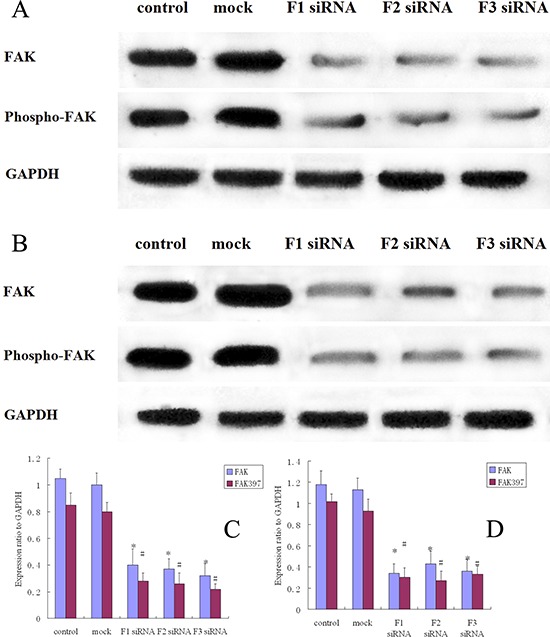
FAK protein expression and FAK phosphorylation in siRNA treated osteosarcoma, control and mock group cells **A.** Western blot analysis of FAK and pFAK in MG-63 cells from control, mock, F1 siRNA, F2 siRNA and F3 siRNA groups. GAPDH was used as a control for protein load and integrity. **B.** Western blot analysis of FAK and pFAK in 143B cells from control, mock, F1 siRNA, F2 siRNA and F3 siRNA groups. GAPDH was used as a control for protein load and integrity. **C.** The bar chart demonstrates the ratio of FAK and pFAK protein to GAPDH by densitometry in MG-63 cells. The data are means ± SEM. **D.** The bar chart demonstrates the ratio of FAK and pFAK protein to GAPDH by densitometry in 143B cells. The data are means ± SEM.

MG-63 and 143B cells were transfected with nonspecific (scrambled) siRNA or FAK siRNA of F1, F2 and F3, harvested and analyzed by Western blot analysis for the presence of FAK protein in order to study the biological function of FAK. Untransfected negative control cells were also analyzed. The FAK protein levels of F1, F2, and F3 siRNA transfected MG-63 cells were significantly reduced by 62%–70%, compared to the untransfected (control group) and Nonspecific siRNA transfected cells (mock group). In addition, the phosphorylation levels of FAK on Tyr397 were significantly lower (a 67%–75% reduction) in the transfected MG-63 cells than in the control and the mock groups (Figure 3). The FAK and pFAK protein levels were similar in the 143B cells (Figure 3).

### The effects of FAK down-regulation on cell proliferation and apoptosis

The effect of decreased FAK expression on osteosarcoma cell proliferation *in vitro* was subsequently examined. The CCK-8 assay results showed that the absorbances (OD value) of MG-63 cells transfected with nonspecific siRNA or F1, F2, F3 siRNA were 0.851 ± 0.065, 0.538 ± 0.059, 0.477 ± 0.053 and 0.499 ± 0.046, respectively. The OD value of untransfected MG-63 cells was 0.880 ± 0.077. The OD values of 143B cells transfected with nonspecific siRNA or F1, F2, F3 siRNA were 0.950 ± 0.105, 0.667 ± 0.060, 0.617 ± 0.059 and 0.598 ± 0.077, respectively. The OD value of untransfected 143B cells was 0.982 ± 0.066. The data were derived from five independent experiments. There were no significant differences between the OD values of the untransfected and nonspecific siRNA transfected cells in the MG-63 and 143-B cell lines (Table 4) (*P* > 0.05).

**Table 4:** The effect of FAK siRNA transfection on cell proliferation

OD Value	Control	Mock^a^	F1 siRNA	F2 siRNA	F3 siRNA
MG-63	0.880 ± 0.077	0.851 ± 0.065	0.538 ± 0.059	0.477 ± 0.053	0.499 ± 0.046
143-B	0.982 ± 0.066	0.950 ± 0.105	0.667 ± 0.060	0.617 ± 0.059	0.598 ± 0.077

a nonspecific (scrambled) siRNA transfected cells

**Figure 4:**
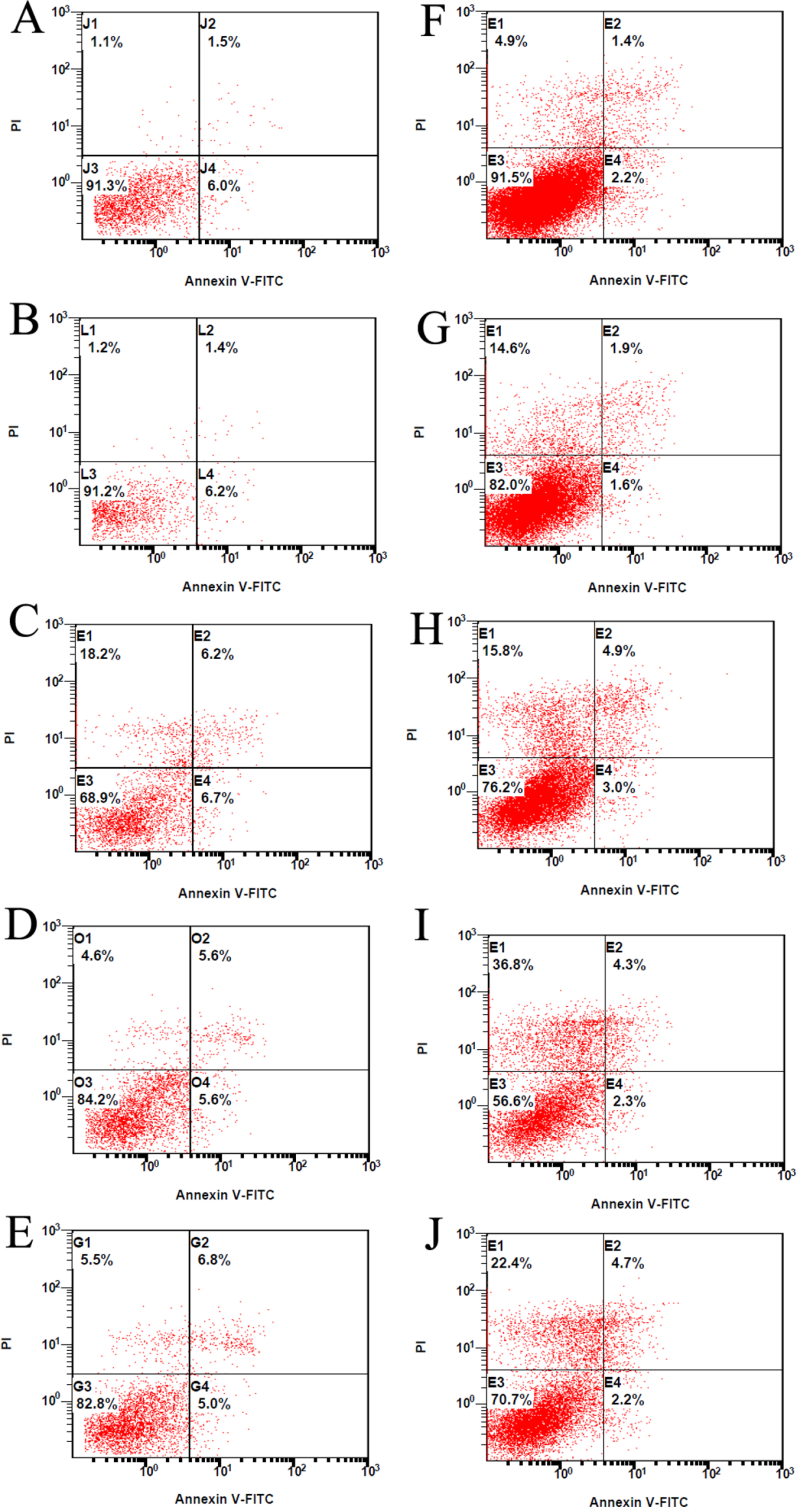
The effect of FAK expression inhibition on osteosarcoma cell apoptosis The MG-63 cells treated with the vehicle control **A.** nonspecific (Scrambled) siRNA **B.** or FAK siRNA of F1 **C.** F2 **D.** and F3 **E.** were analyzed by flow cytometry after staining with Annexin V-FITC/PI. The 143B cells treated with the vehicle control **F.** nonspecific (Scrambled) siRNA **G.** or FAK siRNA of F1 **H.** F2 **I.** and F3 **J.** were analyzed by flow cytometry after staining with Annexin V-FITC/PI.

Alteration of cell apoptosis via FAK knock-down was also measured. The MG-63 and 143B cells were transfected with nonspecific or F1, F2, F3 siRNA and incubated for 24 hours. The cells were then harvested and pelleted by centrifugation for Annexin V/PI staining. Flow cytometry was used to quantify the number of apoptotic cells. The percentage of MG-63 apoptotic cells in the control, mock, F1, F2 and F3 groups was 7.31 ± 0.35%, 7.51 ± 0.45%, 11.89 ± 0.71%, 11.47 ± 0.59% and 12.14 ± 0.72, respectively. The percentage of 143B apoptotic cells in the control, mock, F1, F2 and F3 groups was 3.51 ± 0.27%, 3.62 ± 0.36%, 7.10 ± 0.52%, 6.87 ± 0.42% and 7.21 ± 0.38%, respectively. All experiments were performed five times. These results indicate that FAK siRNA transfection induced osteosarcoma cell apoptosis and reduced cell proliferation (Table 5) (Figure 4).

**Table 5:** The effect of FAK siRNA transfection on cell apoptosis

Apoptosis rate (%)	Control	Mock^a^	F1	F2	F3
MG-63	7.31 ± 0.35	7.51 ± 0.45	11.89 ± 0.71	11.47 ± 0.59	12.14 ± 0.72
143B	3.51 ± 0.27	3.62 ± 0.36	7.10 ± 0.52	6.87 ± 0.42	7.21 ± 0.38

a nonspecific (scrambled) siRNA transfected cells

### Decrease of FAK reduced the migration and invasion of osteosarcoma cell lines

**Figure 5:**
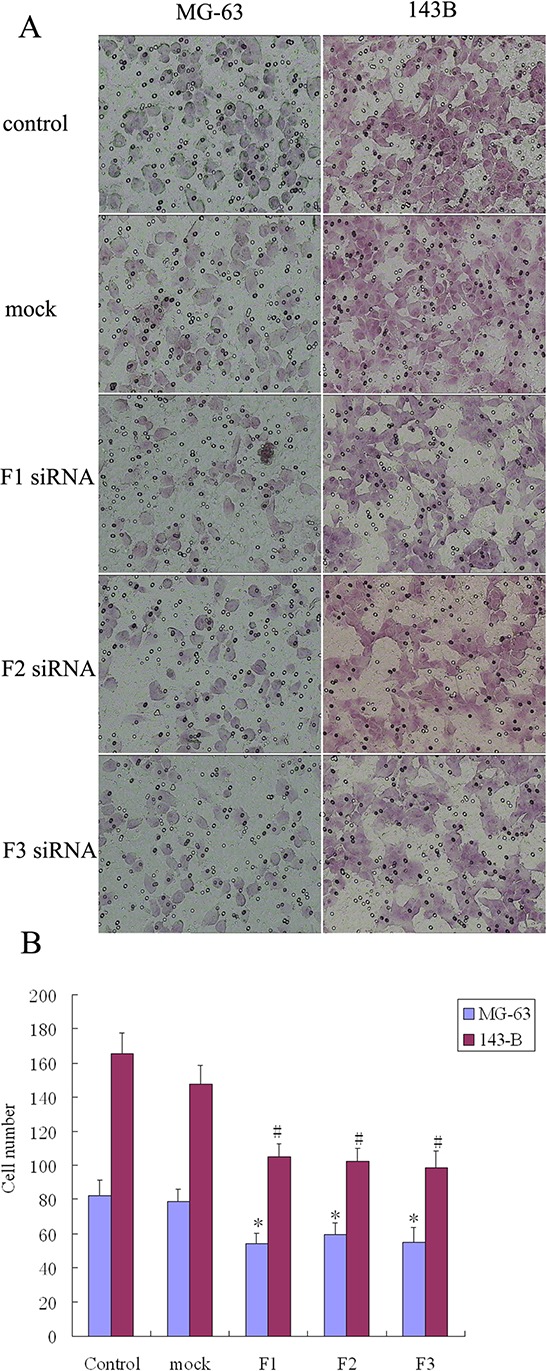
The effect of FAK expression inhibition on osteosarcoma cell migration **A.** Knockdown of FAK inhibited cell migration by transwell assays. The penetration rate through the membrane was higher with untransfected cells (control group) and nonspecific (Scrambled) siRNA transfected cells (mock group) compared with FAK/RNAi cells (200 × magnification). **B.** The number of cells that had migrated to the undersurface of the membrane was counted in 6 fields. Bars, SD. *N* = 5, *P < 0.05.

**Figure 6:**
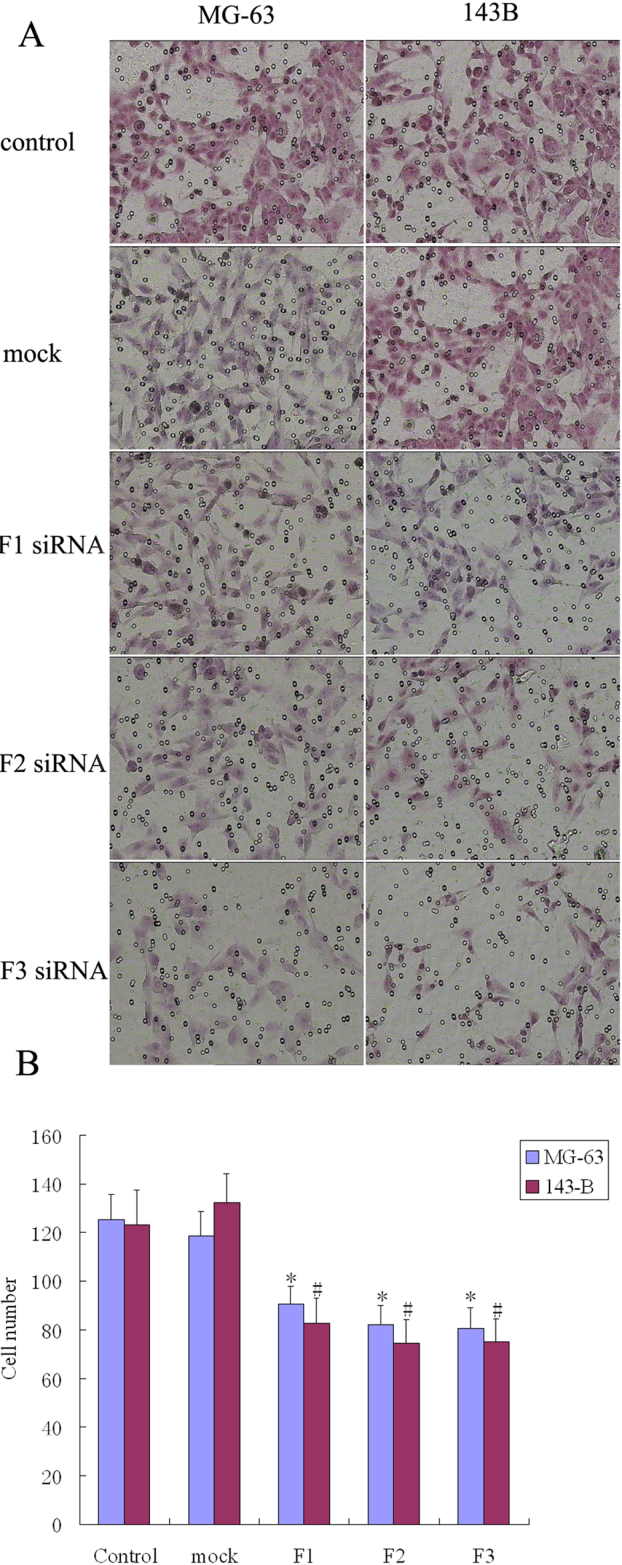
The effect of FAK expression inhibition on osteosarcoma cell invasion **A.** Knockdown of FAK inhibited cell invasion by transwell assays. The invasion ability of untransfected cells (control group) and nonspecific (Scrambled) siRNA transfected cells (mock group) was higher than that of FAK/RNAi cells (200 × magnification). **B.** The number of cells that passed through the membrane was counted in 6 fields. Bars, SD. *N* = 5, *P < 0.05.

The transwell system was used to assay the migration and invasion of transfected MG-63 and 143B cell lines. The number of cells that migrated to the transwell membrane in the siRNA transfection group (F1, F2, or F3) was significantly lower than in the control or mock group (Figure 5). The number of osteosarcoma cells that invaded the membrane was significantly lower when comparing the siRNA transfection group (F1, F2, or F3) to the control and mock groups (Figure 6). These results suggest that FAK overexpression and FAK phosphorylation play important roles in the migration and invasion of osteosarcoma cells.

## DISCUSSION

Several osteosarcoma prognostic markers have been identified, such as ErbB-2 [20], Interferon-a/b receptor [21] and Hsp72 [22]. However, due to a lack of general consensus regarding their prognostic value, most of these factors are rarely taken into consideration when designing treatment regimens and none are currently used to stratify patients before starting therapy [23]. FAK is a known key mediator of cell behavior. Furthermore, autophosphorylation of FAK at Tyr397 has been shown to be a pivotal event in the activation of FAK-mediated cellular functions in response to a variety of extracellular stimuli [13]. Activation and autophosphorylation of FAK leads to its association with several signaling molecules, which then triggers signal transduction to modulate a diverse array of signaling events, which regulate cell adhesion, spreading, proliferation, survival, migration and differentiation [24–25]. Such functions of FAK, recapitulated in tumor cells, are believed to promote tumor growth, survival, migration, invasiveness, metastasis and angiogenesis [26]. Given the important role of FAK in cell adhesion, cell migration and signal transduction, which are of major importance for tumor invasion and metastasis, it seemed reasonable to examine FAK expression in osteosarcoma and the effect of FAK expression on patient outcome. Increased expression of FAK has been described in a variety of human malignancies such as cancer of the liver, lung, and cervix and has been correlated with poor patient outcome [19, 27–30]. However, to the best of our knowledge, there is a lack of direct *in situ* evidence for FAK overexpression and activation in relation to clinicopathological parameters and patient survival in osteosarcoma. Therefore, the role of FAK in osteosarcoma and the correlation between FAK expression and patient survival was the key question we asked in this study. Whether or not FAK activity is required for osteosarcoma cell proliferation, apoptosis, migration and invasion, *in vitro*, was also investigated.

Our data demonstrated that both FAK and pFAK are overexpressed in osteosarcoma compared with normal bone tissue. Although few of the osteosarcoma specimens (*n* = 113) investigated were completely negative for FAK and pFAK, clear differences in staining intensity reflecting the amount of FAK and pFAK were seen overall. These results provide definitive evidence that FAK is elevated and activated in human osteosarcoma tumors. However, the rate of pFAK overexpression in tumors was lower than that of FAK overexpression. The reason for this may be that the presence of proteins that are physically associated with activated FAK may mask the antigen that reacts with anti-pFAK, and leading to an apparent lack of immunoreactivity [10, 31]. Most importantly, FAK expression profiles were found to be significantly related to patient overall survival and metastasis-free survival in the univariate analysis. Specifically, a significant difference in prognosis was found among the 3 different FAK/pFAK coexpression statuses. Subjects that were FAK-/pFAK- had the best outcome, whereas the subjects that were FAK+/pFAK+ had the worst. The considerable differences among the FAK expression profiles suggest an important functional role of FAK and FAK phosphorylation in osteosarcoma progression. Moreover, the FAK/pFAK coexpression profile was found to be an independent prognostic indicator for osteosarcoma. Therefore, FAK and phospho-FAK might be a potential diagnostic tool for differentiating low-risk from high-risk osteosarcoma patients.

Results of the current study also indicate that elevated levels of FAK and pFAK result in enhanced cell migration and invasion in 143B and MG-63 human osteosarcoma cells. Two ATP-competing FAK inhibitors, PF-562271 and TAE226, were found to reduce its kinase activity and tyrosine autophosphorylation. These two inhibitors were also reported to impair tumor cell migration, proliferation, invasion and metastasis *in vitro* and *in vivo* [32–33], thus supporting the importance of FAK activation and signaling in tumor development and progression. To date, most evidence suggests that FAK overexpression and FAK phosphorylation are markers for invasive and metastatic tumors, including carcinomas of the gastrointestinal tract, colon, thyroid, ovary, prostate and oral cavity [17–18, 34–36]. Studies show that metastasis is a process that requires migration and invasion of tumor cells driven by the regulated formation of adhesive structures like focal adhesions (FAs) and invasive structures like invadopodia [37]. Specifically, the attachment of cells to ECM proteins is mainly mediated by integrins, which are heterodimeric transmembrane receptors that connect the ECM to the cellular actin cytoskeleton through FAs [38]. Integrin clustering promotes the formation of cell-matrix adhesions and activation of Src and FAK [39]. When cell migration begins, FAK is localized in cell-matrix adhesions and acts as a signaling center mediating multiple dynamic protein-protein interactions and consequently regulating the assembly and disassembly of FAs [40–41]. In order to achieve this goal, tyrosine phosphorylation of FAK and integrin molecules create docking sites for other proteins involved in actin cytoskeleton remodeling [42]. As a result, in some tumor cell lines, migration has been found to be directly proportional to FAK concentrations [37, 39, 40, 43]. Cell-cell adhesions also undergo a variety of changes when cells metastasize. The level of E-cadherin plays a major role in cell-cell junction strength, and Canel et al. thought that the Src/FAK signaling axis may inhibit the collective movement of tumor cells by controlling E-cadherin internalization [44–45]. This indicates that the Src/FAK signaling axis may play an important role in the cross talk between integrin- and E-cadherin-dependent adhesions in tumor cell migration [46]. In addition, invasiveness is considered to be a vital capability for tumor cells to disseminate locally and metastasize [47]. There have been some reports linking both uPA/uPAR and MMPs, proteolytic enzymes, known to be important for invasion, with FAK activation and signaling in several tumor cell types [48–51]. For example, the cellular arrangement of MT1-MMP is disrupted when the Src-FAK signaling pathway is impeded, which lowers both the expression of MT1-MMP and the activity of MMP-2. This indicates that MMP activation at the cell surface occurs downstream of the Src-FAK signaling pathway [39].

In this study, a clear trend toward decreased overall survival and metastasis-free survival was observed in patients with tumors expressing high amounts of FAK protein and pFAK. Importantly, these findings indicate that disruption of FAK signaling by RNAi has a significant effect on invasion and migration of MG-63 and 143B osteosarcoma cells. Taken together, our results suggest that FAK may promote osteosarcoma cell migration by promoting FAs turnover and modulating FAs dynamics, which is a continuous process involving coordination between FA and the actin cytoskeleton. Based on this, FAK can organize the leading edge by controlling the spatiotemporal variability of osteosarcoma cell protrusion and retraction. Furthermore, MMPs may act as key enzymes responsible for the degradation of the pericellular ECM at the leading edge of the migrating osteosarcoma cells, which is also an event downstream of FAK, resulting in osteosarcoma invasion and metastasis. In fact, FAK inhibition has been found to suppress tumor cell migration and invasion in a variety of human malignancies, such as gastric, lung, oral and ovarian cancer, as well as in melanoma, hepatoblastoma and neuroblastoma [52–60]. These results support the observation that signal transduction pathways initiated by FAK play an important role in mediating tumor cell migration and invasion. Therefore, FAK overexpression and FAK phosphorylation might be a hallmark of osteosarcoma cells with a more aggressive phenotype.

FAK was previously shown to be related to cell survival and cell motility. The survival signal generated in adhered cells is integrin dependent. When epithelial cells are transfected with constitutively activated mutants of FAK, they are rescued from apoptosis under anchorage-independent conditions [61]. Anoikis is a type of apoptosis that occurs when a cell detaches from its supportive matrix. A unique characteristic of tumor cells is resistance to anoikis or the ability to survive and grow in the absence of anchorage to the ECM [62], which depends on the presence of the major autophosphorylation site and kinase activity of FAK [10]. For example, survival signals from cell-ECM interactions are lost when detachment, however the increase in FAK activity may compensate for this loss allowing the lung tumor cells to survive in suspension. Liu et al. reported that Src plays a role in cell resistance to anoikis by interacting with FAK and that Src appeared to be the most important downstream effecter of FAK for mediating cell survival signals [63]. As a result, tumor cells are protected from anoikis by FAK's promotion of the PI3K/AKT and MAPK-ERK pathways, and possibly the MAPK-p38 pathway [63]. In the present study, FAK siRNA had a significant inhibitory effect on cell proliferation and induced cell apoptosis in MG-63 and 143B osteosarcoma cells. Wang et al. showed that inhibition of focal adhesion kinase induced apoptosis in human osteosarcoma SAOS-2 cells [64]. This was similar to our conclusion. Taken together, FAK and FAK phosphorylation might play an important role in cell survival and motility, promoting osteosarcoma development. It is possible that FAK expression is also up regulated and activated by osteosarcoma to promote tumor progression from anchorage-dependent to anchorage-independent growth and metastasis.

Outcomes for patients with osteosarcoma improved dramatically in the 1970 s and 1980 s. The use of combination chemotherapy and surgery enables long-term survival in more than 50% of cases. However, there have been only minimal improvements in the prognosis of osteosarcoma patients over recent decades and the results of osteosarcoma treatment of the extremities have reached a plateau [3, 4]. Therefore, effective novel therapeutic target(s), such as FAK, are highly necessary. Recent studies have identified an inhibitor of FAK autophosphorylation, known as Y15 (1,2,4,5-Benzenetetraamine tetrahydrochloride). In neuroblastoma cells, Y15 blocks FAK phosphorylation at Tyr397 and has been reported to inhibit tumor growth in a variety of tumors [65–67]. Y15 has also been found to enhance inhibition of tumor growth when given in combination with 5-FU and oxaliplatin [67]. In fact, one study suggested that the mechanism by which Y15 leads to apoptosis and necrosis in tumor cells may be synergistic with current and investigational therapeutics [66]. The above results indicate that that FAK may be a potential therapeutic target for osteosarcoma treatment. Src/FAK signaling has been shown to induce E-Cadherin internalization and promote motility of tumor cells, in cases of malignant tumor progression [68–69]. Dasatinib is an oral multi- BCR/ABL and Src family tyrosine kinase inhibitor, which is used to treat patients with chronic myelogenous leukemia (CML) [70–71] or Philadelphia chromosome-positive acute lymphoblastic leukemia (Ph+ALL) [72]. Hence, targeting Src/FAK and associated kinases with dasatinib may also be a useful therapeutic approach in cases of osteosarcoma. This, together with results of the current study, suggest that osteosarcoma development may be inhibited by targeting these kinases, therefore novel therapeutic approaches, designed to block these kinases, may be useful for preventing early tumor invasion and/or development or recurrence of osteosarcoma.

Encouraging results have also been shown by targeting specific protein-protein of FAK interactions in a variety of cancer models [73–75]. Vascular endothelial growth factor receptor 3 (VEGFR-3) is a major component of the FAK scaffold. Targeting the protein-protein interaction site would be a novel approach to FAK inhibition, along with direct disruption of downstream signaling. FAK and VEGFR-3 proteins, as well as their complexes are present in tumors and inhibition of FAK and VEGFR-3 will affect signaling in tumors and tumor microenvironments, thus both are promising therapeutic targets. A recently identified molecular inhibitor, C4 (chloropyramine hydrochloride), has been found to disrupt the survival functions of VEGFR-3 and FAK by targeting the interaction site of VEGFR-3-FAK [73, 76]. In previous studies, C4 acted synergistically with doxorubicin chemotherapy in breast cancer xenograft models and markedly reduced the growth of breast tumors [73, 76]. Furthermore, when combined, low (nanomolar) doses of both C4 and GEM affected cell viability and induced apoptosis, synergistically [76]. Therefore, novel osteosarcoma treatments may also be developed by disrupting the FAK scaffolding function using small-molecule inhibitors.

In summary, findings in the current study indicate a correlation between osteosarcoma malignancy *in vitro* and *in vivo* and FAK overexpression and FAK phosphorylation. This suggests that FAK plays an important biological role in osteosarcoma carcinogenesis and provides a better understanding of the diagnostic and prognostic relevance of FAK overexpression and FAK phosphorylation in osteosarcoma. Therefore, FAK and pFAK may be useful independent predictors of overall survival and metastasis-free survival in osteosarcoma patients. Novel therapeutic approaches could be used to alter FAK activity, and the resulting FAK function, for the treatment of osteosarcoma.

## MATERIALS AND METHODS

### Patients and tissue samples

Paraffin-embedded conventional osteosarcoma tissue samples were obtained from the Tumor Tissue Bank of The Third Affiliated Hospital of Soochow University and Jinling Hospital, between 1999 and 2009. Certified pathologists verified the diagnosis of osteosarcoma.

The inclusion criteria for this study were as follows: 1) extremity osteosarcoma, 2) American Joint Committee on Cancer (AJCC) surgical stage II, 3) scheduled for preoperative neoadjuvant chemotherapy and curative surgery followed by adjuvant chemotherapy at our institute, 4) osteoblastic subtype and 5) presence of incisional biopsy specimens available for immunohistochemistry. The exclusion criteria were as follows: 1) secondary osteosarcomas, 2) extraskeletal osteosarcomas, 3) periosteal or paraosteal osteosarcomas, 4) patients with skip lesions or metastatic disease at diagnosis, 5) patients who had received prior chemotherapy or radiation, and 6) patients lost to follow-up.

Informed consent was obtained from all patients or legal guardians prior to inclusion in the study, as appropriate. The institutional research review board at Soochow University approved this study in accordance with the ethical standards laid down in the 1964 Declaration of Helsinki. All patients underwent preoperative chemotherapy, surgery and postoperative chemotherapy according to the IOR/OS-2 protocol [77], which was modified for each patient based on his or her general condition, past history of chemotherapy, and vital organ function. In the initial evaluation, the primary tumor was assessed by conventional radiographs, a technetium 99-MDP bone scan, a CT and MRI (after 2002) scan of the entire bone involved, and additional chest plain radiographs and CT scans. During chemotherapy, patients were examined every 2 months via chest CTs and radiographs of the operated limb. After completion of chemotherapy, follow up was done on each patient every three months for the first three years with X-rays of the tumor site, a CT or MRI of the tumor site and a CT of the chest. During the fourth and fifth years, follow up was done, as above, every 6 months and then annually. Bone scans were conducted annually. Careful physical examinations were also performed at each visit. When any suspicious sign or symptom related to local recurrence or distant metastasis appeared, patients could contact and visit us immediately. If necessary, additional radiographic examinations were performed on suspicious areas after physical examination.

Clinicopathologic variables were recorded including age, gender, tumor location, AJCC surgical stage, surgical type (amputation or limb salvage surgery) and histological response to pre-operative chemotherapy (tumor necrosis rate). Histologic responses to pre-operative chemotherapy were assessed at the time of surgery and recorded as the percentage of tumor necrosis. The clinical outcome was followed from the date of surgery until the date of death or until December 2013. Cases that died of other causes and cases that were alive at the end of follow-up were regarded as censored data for the survival analysis. Survival time was defined as the time span from diagnosis until death due to primary tumor-related causes.

### Main reagents

Primary antibodies included rabbit polyclonal antibody against human FAK (C-20) (1:100 dilution; Clone No. sc-558, Santa Cruz Biotechnology, CA, USA) and rabbit monoclonal antibody against human phosphorylated FAK[pY397] (1:100 dilution; Clone No. 700255, Invitrogen, Carlsbad, CA, USA). Heat-induced epitope retrieval in citrate buffer (0.01 mol/L; pH 6.0) was applied to all slides before immunohistochemical staining, as described below. Immunostaining was performed with an Envision™ detection system kit (Dako, Glostrup, Denmark). CCK-8 kits (Kaiji, Nanjing, China) were used to assay cell proliferation. Cells were transfected using Lipofectamine 2000 (Invitrogen). BCA Protein Assay Kit from Kaiji was used to measure protein concentration. The other chemicals and reagents used were of analytical grade.

### Immunohistochemistry

FAK-staining by immunohistochemistry was performed using antibodies against FAK and FAK[pY397] (pFAK), according to manufacturer's instructions, after preliminary screening on H&E stained slides. Sections of 4 μm-thick, formalin-fixed, paraffin-embedded osteosarcoma tissue samples were mounted on poly-L-lysine-coated slides. Slides were deparaffinized in xylene. Endogenous peroxidase activity was blocked with 3% hydrogen peroxide in 50% methanol for 10 min at room temperature. Sections were rehydrated in alcohol, washed with phosphate-buffered saline (PBS) and then pretreated with citrate buffer (0.01 M citric acid, pH 6.0) for 20 min at 95°C in a microwave oven. After blocking of nonspecific binding sites by exposure to 10% normal goat serum in PBS for 20 min at 37°C, sections were incubated overnight, at 4°C, with rabbit polyclonal antibody against human FAK or rabbit monoclonal antibody against human pFAK, followed by immunodetection using the EnVision™ + System. The slides were rinsed with distilled water for 5 min, counterstained with Mayer's hematoxylin for 1 min, dehydrated through an alcohol gradient and sealed with cover slips. Appropriate positive and negative controls were tested in parallel.

To quantify FAK and pFAK expression, an experienced board-certified, masked pathologist scored the intensity of staining in tissue sections as follows: 0 (none), 1 (borderline), 2 (weak), 3 (moderate) and 4 (strong). The percentage of positive cells was also calculated, as described previously [78–79]. Samples with staining intensities ≥3 and percentages of positive cells ≥90% were considered to have high FAK or pFAK expression [78–79].

### Cell culture

Human osteosarcoma cell lines, 143B and MG-63, were obtained from American Type Culture Collection (Manassas, VA, USA) and were cultured in Dulbecco's Modified Eagle Medium (DMEM; Invitrogen), supplemented with 10% fetal bovine serum (FBS, Invitrogen), penicillin (100 U/ml) and streptomycin (100 mg/ml; Invitrogen), at 37°C in a 5% CO2 incubator. All cells used in the experiments were in the exponential growth phase.

### Transfection of siRNA

Four siRNA oligonucleotides were designed as previously described [80–83] and synthesized by Qiagen GmbH (Hilden, Germany) to target the following cDNA sequences: Nonspecific (Scrambled) siRNA, 5′-AATTCTCCGAACGTGTCACGT-3′; FAK siRNA, F1: 5′-GGUUCAAGCUGGAUUAUUU-3′, F2: 5′-CCGGTCGAATGATAAGGTGTA-3′, F3: 5′- GGAAAUACAGUUUGGAUCU-3′. The 143B and MG-63 cells were then transfected with either FAK specific (F1, F2 or F3, as specified in text) or nonspecific (scrambled) siRNA using Lipofectamine 2000, according to the manufacturer's instructions.

### Western blot

The MG-63 and 143B cell layers were washed with ice cold PBS and lysed in a lysis buffer for 20 min at 4°C. The protein concentration in each cell lysate was then measured using a commercial BCA kit. A 50 μg sample of each lysate was fractionated on an 8% SDS-PAGE, stacked at 80 V for 30 min, separated at 120 V for 1 h and transferred to PVDF membranes at 15 V. Membranes were blocked for 1 h at room temperature with 5% nonfat-milk and incubated with different primary antibodies at 4°C overnight. Membranes were then incubated with horseradish peroxidase (HRP)-labeled secondary anti-rabbit IgG antibody for 2 h at room temperature. Image pro plus (IPP) software for densitometry analysis was used for the quantification of protein expression.

### Cell proliferation assay

The MG-63 and 143B cells, transfected with or without siRNA, were plated in 96-well plates in DMEM medium containing 10% FBS at a density of 1 × 10^5^ cells/mL and incubated for 24 h. A 10-μL volume of the CCK-8 solution was then added to each well and the cells were cultured for another 4 h. The plate was then read in a spectrometer at 450 nm to determine the absorbance of each well (OD value).

### Apoptosis assay

MG-63 and 143B cells were plated on 6-well plates at a density of 2 × 10^5^ cells/well to assess cellular apoptosis. Cells were incubated for 24 h and then harvested and pelleted by centrifugation for Annexin V/PI staining. For the assessment, cell pellets were washed twice with phosphate buffer and re-suspended in 250 μL binding buffer at a density of 1 × 10^6^ cells/mL. The cells were stained with 5 μL Annexin V-FITC and 10 μL PI solution (20 μg/mL), incubated on ice for 15 min and 100 μL of the cell suspension was analyzed by flow cytometry to detect apoptosis.

### Cell migration and invasion assay

The cell migration and invasion assay was performed using a Transwell system, according to the manufacturer's protocol (BD Biosciences). The MG-63 and 143B cells were trypsinized and pelleted by centrifugation. The cells were washed twice in phosphate buffer, re-suspended in serum free DMEM medium at a density of 2 × 10^5^ cells/ml and 200 μL of the cell suspension was seeded in the upper chamber of a 24-well transwell for the migration assay. Matrigel (80 μL) (BD Biosciences) was added to the upper chamber of the 24-well transwell, the transwell was incubated at 37°C for 60 mins to form a matrix gel and the cells were seeded in the coated chamber for invasion assay. A volume of 500 μL DMEM medium supplemented with 5% FBS was subsequently added to the lower chamber of a 24-well transwell migration system. Twenty four hours later (48 hours for the invasion of MG-63), the inserts were removed, the cells and gel on the upper surface were scraped using a cotton swap and the cells were fixed with 4% Paraformaldehyde (PFA) for 20 minutes at room temperature, prior to Hematoxylin staining. Stained inserts were subsequently cut and mounted on microscope slides. Digital slides were taken using a digital microscope (200 fold) and 6 fields were counted from each insert. The results of five independent experiments were averaged.

### Statistical analysis

SPSS version 11.5 (SPSS, Chicago, IL, USA) was used for all statistical calculations. Values are shown as mean/median ± SD or as percentages. For statistical analysis, the χ2 test was used to compare categorical data and the *one-way ANOVA* test was used to compare quantitative data. Survival rates were calculated using the Kaplan-Meier method and statistically significant differences were identified using the log-rank test. Patients who died of diseases other than osteosarcoma were treated as censored cases. Multivariate analyses were carried out to identify independent prognostic factors for survival using the Cox proportional hazards model. The significance level was set at 0.05 for each analysis.
